# Climate change-related concerns in psychotherapy: therapists’ experiences and views on addressing this topic in therapy

**DOI:** 10.1186/s40359-024-01677-x

**Published:** 2024-04-08

**Authors:** Katharina Trost, Verena Ertl, Julia König, Rita Rosner, Hannah Comtesse

**Affiliations:** Clinical and Biological Psychology, Catholic University Eichstaett-Ingolstadt, Eichstaett, Germany

**Keywords:** Climate change, Psychotherapists, Mental health treatment, Climate anxiety

## Abstract

**Background:**

While adverse impacts of climate change on physical health are well-known, research on its effects on mental health is still scarce. Thus, it is unclear whether potential impacts have already reached treatment practice. Our study aimed to quantify psychotherapists’ experiences with patients reporting climate change-related concerns and their views on dealing with this topic in psychotherapy.

**Methods:**

In a nationwide online survey, responses were collected from 573 psychotherapists from Germany. Therapists reported on the presence of such patients, their socio-demographic characteristics, and climate change-related reactions. Psychotherapists’ views on dealing with this topic in psychotherapy were also assessed. Descriptive statistics were used to analyse the responses.

**Results:**

About 72% (410/573) of psychotherapists indicated having had patients expressing concerns about climate change during treatment. Out of these therapists, 41% (166/410) stated that at least one patient sought treatment deliberately because of such concerns. Patients were mainly young adults with higher education. Most frequent primary diagnoses were depression, adjustment disorder, and generalized anxiety disorder. Psychotherapists having encountered such patients differed from those without such encounters in their views on potential functional impairment and the necessity to target the concerns in treatment. Although 79% (326/415) of all respondents felt adequately prepared by their current therapeutic skills, 50% (209/414) reported a lack of information on how to deal with such concerns in therapy.

**Conclusions:**

Results indicate that psychotherapists are frequently confronted with climate change-related concerns and regard the mental health impact of climate change on their patients as meaningful to psychotherapeutic care. Regular care could be improved by a continuous refinement of the conceptualization and knowledge of the mental health influences of climate change. This would allow providing tailored methods of assessing and addressing climate change-related concerns in practice.

**Supplementary Information:**

The online version contains supplementary material available at 10.1186/s40359-024-01677-x.

## Introduction

Consequences of climate change are affecting an increasing number of people around the world [1]. While the ways in which climate change impacts physical health have been recognized for some time (e.g., 2), mental health outcomes have become a focus in recent years [3]. Results show that both acute and chronic (anticipated) consequences of climate change can affect mental health via diverse pathways [4–6]. Recent reviews on climate change impacts on mental health have highlighted a potential relation between acute climate change consequences and mental disorders [6–9]. It is well established that acute events, such as floods or wildfires, are associated with traumatic stress. For example, Kessler, Aguilar-Gaxiola [10] showed in a review that single event natural disasters were one of the 29 trauma event types with an increased risk of posttraumatic stress disorder (PTSD). Furthermore, Neria, Nandi [11] reported in a systematic review PTSD prevalence rates between 4% and 60% after natural disasters such as earthquakes, floods, hurricanes, and wildfires around the world from 1963 to 2005, depending on degree of exposure (e.g., proximity to epicenter, extent of disruption) and sample characteristics. While the association between acute events and PTSD is well established, reviews also proposed a relationship between acute weather events and an increase in anxiety disorders and depression [5–8, 12]. Although not every weather extreme or disaster is caused by climate change, it is an established fact that natural disasters are becoming more frequent with climate change progressing [13]. Consequently, the possible negative effects of climate change on mental health are likely to increase.

The chronic effects of climate change (e.g., drought) on mental health are more difficult to operationalize, because impacts are mostly indirect and delayed, and factors interact in multiple ways (e.g., [14], for drought, [15]). However, recent (narrative) reviews on climate change impacts on mental health did focus on chronic influences (e.g., drought, increase in temperature and sea-level, deforestation) on mental health [5–7, 12, 16]. The review conducted by Palinkas and Wong [6], for example, assumed that subacute consequences of climate change (e.g., heat waves) can exacerbate existing mental disorders (e.g., substance abuse disorders due to diminished thermoregulation). Additionally, this review found that experiencing drought episodes (primarily studied in Australia) is associated with generalized anxiety disorder and depression, among other symptoms, due to factors such as economic effects and migration.

Furthermore, besides the exposure to climate change-related disasters and the perception of chronic climate change hazards, research has suggested that the awareness of the existential threat of climate change (e.g., ecological losses), evokes emotional reactions and may affect mental health (e.g., [5]). To capture these reactions, new concepts of climate/eco-emotions such as climate change anxiety [17] and ecological grief [18] have been introduced. However, these concepts have only recently been quantified [17, 19], with first results indicating associations of severe levels of these concepts with functional impacts in daily life (e.g., [20]). At the general population level, several large-scale studies have shown that significant numbers of people in different countries are emotionally affected by worries, fears and sadness about environmental changes attributed to climate change [20–22]. For example, in a representative German survey conducted in 2022 more than a half of the participants (55%) indicated to be sad about natural destruction, around a quarter (23%) fully agreed with the statement “I am afraid of the consequences of climate change”, and almost a quarter of people (22%) felt psychologically stressed by climate change and environmental destruction, 5% of whom felt very stressed [22].

Taken together, these findings suggest that concerns about climate change seem to be widespread and may also become evident in clinical groups [23–25]. A recent study conducted in the USA investigated the experience, attitude, and knowledge of mental health professionals (MHP, *N* = 517) with regard to the impact of climate change on mental health and its effect on treatment [24]. The majority of participants (57%) strongly agreed that the consequences of climate change influence mental health. Additionally, 54% of MHPs indicated that they had already seen clients who raised climate change-related concerns during treatment. MHPs reported that these concerns were related to symptoms of generalized anxiety, depression, grief reactions, and post-traumatic stress in these clients. Further, the majority of MHPs stated that they lacked tools for assessment and treatment as well as information on referral possibilities for these clients. Similar results were yielded in a sample of physicians and nurses in the USA [25]. However, it remains unclear whether and to what extent this is also the case in psychotherapeutic care in Europe.

The current study aimed at examining whether German psychotherapists are currently already encountering patients with climate change-related concerns in their practice. Therefore, we recruited a nationwide sample of psychotherapists working in different settings in Germany as possible for an online survey and queried them about these concerns as well as gathering information on patients’ demographics and clinical status. Further, we explored cognitive, emotional, physiological, and behavioral reactions of their patients regarding the respective climate change concerns. Finally, we examined psychotherapists’ views on dealing with climate change-related concerns during treatment. In this regard, we investigated whether psychotherapists who had encountered patients with concerns (therapists with experience) differed in their views from those who had not yet encountered such patients (therapists without experience).

## Method

### Participants and procedure

The study was conducted as a nationwide cross-sectional online survey among psychotherapists, both licensed and in training, across all therapeutic approaches recognized in Germany. The therapeutic approaches in Germany are: cognitive behavioral therapy (CBT), psychoanalysis (PA), depth psychology (DP), systemic therapy (ST). Inclusion criteria were (a) being a licensed psychotherapist or psychotherapist in training and (b) giving informed consent to participate in the survey. The study was approved by the ethics committee of the Catholic University Eichstätt-Ingolstadt (number: 122–2022). Data collection took place between February and April 2023.

Our recruitment approach aimed to reflect the reality of the German psychotherapeutic care system as accurately as possible. Therefore, all regional Psychotherapists’ Chambers (“Psychotherapeutenkammern”), in which licensed psychotherapists need to be registered, were asked to forward the online survey link to their members. After a follow-up, commitments from eight out of 12 chambers were received. To include psychotherapists in training we used a random sampling approach. Training institutions in each of the 16 federal states were asked to forward the survey to their trainees. Given the absence of an official comprehensive list of all registered training institutions in Germany, we made a concerted effort to compile a thorough inventory of training institutes across the federal states (up to January 2023). This was achieved by utilizing the websites of psychotherapist chambers (Bavaria, Berlin, Bremen, Hessen, Niedersachsen, Nordrhein-Westfalen, Saarland, Schleswig-Holstein). In cases where lists were outdated or unavailable, additional searches were conducted on the official websites of states (Baden-Württemberg, Brandenburg, Hamburg, Mecklenburg-Vorpommern, Rheinland-Pfalz, Sachsen) as well as the German Association of Psychotherapists (DPtV, Sachsen-Anhalt. Thüringen). The final list comprised 271 institutions across all federal states and therapeutic approaches.

In each of the federal states, a random selection of 10% (in total, *n* = 33) of the institutions was contacted to forward the survey to their trainees. In case an institution denied distributing the survey to their trainees, another institution for this federal state was randomly selected. At the end of the recruitment process, a sum of 45 training institutions was contacted, of which 27 distributed the survey link to their trainees. The 10% target could not be achieved in 5 federal states (Bavaria, Bremen, Hessen, Nordrhein-Westfalen, Rheinland-Pfalz). In addition, all regional associations of statutory health insurance physicians (“Kassenärztliche Vereinigungen”) and three professional associations of psychotherapists that operate across therapeutic approaches and throughout Germany (“Berufsverbände”, Association of Psychological Psychotherapists in the professional association of German psychologists, BDP-VPP; Federal Association of Contract Psychotherapists, BVVP; German Association of Psychotherapists, DPtV) were requested to distribute the survey. Two out of three requested professional associations published the survey link on their homepage and five out of 17 regional associations of statutory health insurance physicians (Bremen, Hamburg, Niedersachsen, Westfalen-Lippe, Thüringen) forwarded the survey to their members. Members of the national bodies could be licensed psychotherapists and psychotherapists in training. Members of associations of statutory health insurance physicians were licensed.

All contacted institutions received detailed information about the study by phone and e-mail and distributed the survey information, link, and QR-Code electronically via e-mail, internal newsletter, and/or a notice on their homepage and intranet. Four training institutions placed an announcement (printed version of the tender text) on their bulletin board.

A total of 624 psychotherapists clicked on the survey link, of whom 51 denied consent or did agree and dropped out before answering to the items for experience regarding patients with climate change-related concerns. Thus, we analyzed the responses of the remaining participants (*N* = 573). Dropouts after the consent page were not excluded from subsequent analyses as participants dropped out at different stages of the survey, and itemwise analyses were conducted (see 24, for a similar approach). To ensure the robustness of this approach, we contrasted participants with more and less than 10% missing values across the survey on all items. This yielded no significant differences in terms of therapists' characteristics, experiences with patients with climate change-related concerns, or views on the topic.

### Measures

The survey comprised 37 items, of which 24 items administered to all participants and 13 items (focusing on climate change-specific reactions) presented only to those reporting that they had already treated patients with climate change-related concerns (i.e., therapists with experience). Items were newly developed for this study and based on a large-scale survey on experience, attitude, and knowledge of MHPs with climate change topics raised by their clients [24]. To ensure comprehensibility and relevance of all items, the survey was piloted by five psychotherapists in training before circulation. The survey was provided online using the survey tool Qualtrics. The complete survey is presented in Appendix A.

At the beginning of the survey, socio-demographic and work-related information about the participants was collected in ten items about: age (year of birth), gender (female/male/diverse), level of training (trainee vs. licensed), therapeutic approach (CBT, PA, DP, ST, other), work experience (number of years working as a therapist including time as trainee, number of weekly treatment sessions), and practice setting (private practice, hospital, outpatient clinic, other). Additionally, engagement in climate or pro-environmental advocacy groups was assessed dichotomously. The degree of pro-environmental behavior in everyday life was assessed on a 4-point scale (1 = *in no area of everyday life*, 4 = *in almost all areas of everyday life).* Thereafter, participants were assigned to one of two paths, depending on whether they had already encountered patients expressing climate change-related concerns. Path A (for therapists with experience) collected information about the number of such patients (seen in the last 12 months), the patients’ socio-demographic characteristics as well as their cognitive, emotional, physiological, and behavioral reactions. Predefined answer options for cognitive styles (e.g., rumination), physiological (e.g., racing heart) and behavioral reactions (e.g., crying) were formulated according to our current knowledge of human stress response and the recent literature on climate change-related emotions [17, 19, 26]. In addition, their expression of feelings related to climate change-related concerns were collected in free-text format. In path B, therapists without experience were asked whether they expected to encounter patients with climate change-related concerns in the future. In the first two parts, for most items multiple responses were allowed (therapists’ practice setting and therapeutic approach; patients’ age in years, educational degree, assumed family status, most frequent assigned diagnoses, cognitive styles, feelings, physiological and behavioral reactions). In the last part of the survey, all participants (therapists with and without experience) answered 12 questions regarding their views on climate change-related concerns (a) in relation to mental health, (b) on how to deal with them in therapy and (c) whether they felt well equipped or wished for additional training and resources on the topic. Therapists answered on a 4-point scale (1 = *I do not agree at all*, 4 = *I fully agree).*

### Statistical analysis

All analyses were performed itemwise because the survey did not employ a forced choice format and participants dropped out at different stages of the survey. This means that we included the number of participants who had answered the respective items (indicated by n/N for all frequencies reported; see 24, for a similiar approach).

Descriptive statistics were used to describe items presented in the three parts of the survey, using frequencies or mean values. The free text answers related to the patients’ feelings were mostly given in one word per option (e.g., anxiety, fear, anger). The answers were categorized inductively [27] and analyzed descriptively.

For contrasting therapists with and without experience with regard to their views on climate change-related concerns in therapy, all items presented in the last part of the survey were dichotomized in 0 = *disagreement* (on the 4-point scale: 1 = *I do not agree at all*, 2 = *I rather disagree*) and 1 = *agreement* (on the 4-point scale: 3 = *I rather agree*, 4 = *I fully agree*). Group differences were computed using chi-square test, t-tests, and Mann-Whitney-U-test, depending on the type of data. All tests were two-tailed with α = 0.05. Bonferroni-Holm correction was performed within each thematic group of items asking about therapists’ views (i.e., views on consequences of climate change-related concerns for mental health, views on how to address climate change-related concerns in therapy and views on required resources for addressing climate change-related concerns in therapy). Data were analyzed using SPSS statistics, version 28.

## Results

### Therapists’ socio-demographic information

Psychotherapists’ socio-demographic and work-related information is shown in Table 1. Therapists were on average 49 years old and mostly female (75.6%, 433/571). The sample consisted predominantly of licensed therapists (87.1%, 499/573), who had been working with patients for an average of 15.2 years. Most therapists worked in private practices (79.0%, 453/573), while 11.0% (63/573) were employed in hospitals. Specialized on treating adults (73.6%, 422/573), therapists worked with an average of 19.1 treatment sessions per week over the last year. The most frequent approaches were CBT (56.5%, 324/573) and DP (40.7%, 233/573). Around 80% (450/570) of participants reported behaving climate change-conscious in *many* to *almost all areas of everyday life*. About 17% (99/568) reported being actively involved in “for future-” movements (e.g., “fridays for future”), or other climate or pro-environmental advocacy groups.

**Table 1:** Participants’ socio-demographic and work-related information

**Age in years, M (SD) n** ^†^ ** = 571**	48.5 (12.4)
**Gender, % (*n*)***n*^†^ = 571	
Female	75.6 (433)
Male	22.9 (131)
Divers	1.2 (7)
**Work-related variables**	
**Level of qualification, % (*n*)***n*^†^ = 573	
LPTs (with approbation)	87.1 (499)
PiTs (in training for approbation)	12.9 (74)
**Practice setting, % (*n*)***n*^†^ = 573 (*multiple answers possible*)	
Private practice	79.0 (453)
Hospital	11.0 (63)
Outpatient clinic	12.7 (73)
Other	7.5 (43)
**Weekly therapy sessions**, ***M*(*SD*)***n*^†^ = 573	19.1 (8.8)
**Type of license, % (*n*)***n*^†^ = 573	
Psychotherapist for adults	73.6 (422)
Psychotherapist for children and adolescents	19.4 (111)
Psychotherapist for adults, additional qualification for children and adolescents	4.0 (23)
Physician psychotherapist	1.8 (10)
Other	1.2 (7)
**Working years**, ***M (SD)****n*^†^ = 570	15.2 (10.3)
**Therapeutic approach, % (*n*)***n*^†^ = 573 (*multiple answers possible*)	
CBT	56.5 (324)
DP	40.7 (233)
PA	16.1 (92)
ST	3.3 (19)
Other	3.1 (18)
**Environmental engagement**	
**Engagement in advocacy groups**, **% (*n*)***n*^†^ = 568	17.4 (99)
**Climate-friendly everyday behavior, % (*n*)***n*^†^ = 570	
In almost all areas of everyday life	15.3 (87)
In many areas of everyday life	63.7 (363)
In a few areas of everyday life	18.2 (104)
In no area of everyday life	2.8 (16)

Note. LPT, licensed psychotherapist; PiT, psychotherapist in training; CBT = cognitive behavioral therapy; DP = depth psychology, PA = psychoanalysis, ST = systemic therapeutic approach; *n*^**†**^**=** Number of all participants answering this item

### Therapists’ experience with patients with climate change-related concerns

Experience with patients with climate change-related concerns was reported by 71.6% (410/573) of the participants. Of the therapists without experience, 58.6% (95/163) expected to encounter more patients with climate change-related concerns in the future.

Of the therapists with experience (*n* = 410), 84.9% (348/410) reported having encountered a range of one to 30 of such patients, with 66.6% (273/410) indicating between one and 10 patients expressing climate change-related concerns. Of the therapists with experience (*n* = 410) 364 replied to the question whether. Around 40.5% (166/410) reported that at least one patient with climate change-related concerns had stated that such concerns were the explicit reason for seeking therapy.

Table 2 displays information provided by therapists with experience about socio-demographic characteristics of their patients with climate change-related concerns. Patients with such concerns were described as mainly young (19 to 24 years, 64.0%, 210/328) and early middle aged (25 to 34 years, 57.6%, 189/328) adults, as well as higher educated (higher education entrance qualification, 77.2%, 251/325; university degree, 66.8%, 217/325). Mostly, these patients were living in a relationship (62.3%, 197/316) and without children (53.4%, 171/320). Therapists indicated having diagnosed these patients with mostly depression (53.0%, 167/315), adjustment disorder (12.4%, 39/315), and generalized anxiety disorder (11.1%, 35/315) as the primary diagnoses for seeking treatment.

**Table 2:** Psychotherapist-reported socio-demographic characteristics of patients having expressed climate change-related thoughts and feelings

**Age in years, % (n)** *n* ^†^ *= 328 (multiple answers possible)*	
< 14 years	9.8 (32)
14 to 18 years	23.2 (76)
19 to 24 years	64.0 (210)
25 to 34 years	57.6 (189)
35 to 44 years	39.0 (128)
45 to 59 years	29.9 (98)
60 to 70 years	18.0 (59)
> 70 years	7.0 (23)
**(Intended) educational degree, % (*n*)***n*^†^ = 325 (*multiple answers possible*)	
Basic school certificate	14.5 (47)
Intermediate school certificate	38.2 (124)
higher education entrance qualification (A-Levels)	77.2 (251)
university degree	66.8 (217)
**Assumed family status, % (*n*)***n*^†^ = 316 (*multiple answers possible)*	
majority of such patients is in a relationship	62.3 (197)
majority of such patients is not in a relationship	33.0 (103)
majority of such patients is too young for a relationship	16.5 (52)
**Having children, % (*n)****n*^†^ = 320	
majority of such patients have children	27.8 (89)
majority of such patients do not have children	53.4 (171)
majority of such patients are still a child themselves	18.8 (60)
**Most frequently assigned diagnosis, % (*n*)***n*^†^ = 315 *(asked to sort by frequency)*	
Depression	53.0 (167)
Adjustment Disorder	12.4 (39)
Generalized anxiety disorder	11.1 (35)
Panic disorder	6.7 (21)
Somatoform disorders	6.7 (21)
Posttraumatic stress disorder	5.4 (17)
Agoraphobia	1.9 (6)
Other	4.8 (15)

Note. *n*^**†**^**=** Number of all participants answering this item

Therapists with experience reported a range of cognitive, emotional, physiological, and behavioral reactions of patients with climate change-related concerns. For the detailed list of patients’ reactions see Table A in Appendix B. Rumination was indicated as the most common cognitive style (73.1%, 231/316), besides catastrophic thoughts/ disaster thoughts (59.2%, 187/316). Within the “Other” category, 3.8% (12/316) of the therapists with experience reported effective solution- and action-oriented styles.

Furthermore, therapists with experience indicated that *anxiety* (88.6%, 194/219) with manifestations from worrying to panic, *helplessness* (60.7%, 133/219) with hopelessness and feelings of despair, *anger* (60.3%, 132/219) and *grief* (35.2%, 77/219) including disconsolateness and the feeling of senselessness were the four most frequent reported feelings in their patients.

More than two thirds of the therapists with experience who answered to the questions concerning physiological and behavioral reactions noticed physiological (68.2%, 176/258) and behavioral reactions (83.6%, 219/262). Around 60% (155/258) reported sleep disorders in their patients. Therapists indicated that avoidance (57.3%, 150/262), aggression (44.7%, 117/262), and crying (34.0%, 89/262) were the three most common behavioral reactions in their patients when climate change-related concerns were addressed in therapy.

### Comparison of therapists with and without experience

Results of comparisons on socio-demographic and work-related characteristics between therapists with and without experience are summarized in Table 3. Therapists with experience were significantly more often female and reported more climate friendly everyday behavior and engagement in climate change-related advocacy groups. They also indicated a significantly higher patient-load in the last 12 months. There were no differences in age and therapeutic approach.

**Table 3:** Comparison of psychotherapists with and without experience with patients expressing climate change-related thoughts and feelings in therapy

Variables		Groups (with experience / without experience)	Test statistics
**Socio-demographic data**			
Gender, % (*n*)	female	80.4 (328) / 64.4 (105)	*χ*^*2*^(2) = 16.22, *p <* 0.001***
	male	18.6 (76) / 33.7 (55)	
Age, *M* (*SD*)		48.9 (12.2) / 47.6 (13.0)	*t* (569) = 1.14, *p* = 0.256
**Engagement-related variables**			
Climate friendly everyday behavior, *M* (*SD*)		3.0 (0.6) / 2.7 (0.8)	*z* = -4.56, *p <* 0.001***
Engagement in advocacy groups, % (*n*)	yes	20.2 (83) / 9.8 (16)	*χ*^*2*^ (1) = 8.98, *p =* 0.003**
	no	78.8 (323) / 90.2 (147)	
**Work-related variables**			
Therapeutic approach (multiple answers possible), % (*n*)	CBT	56.1 (230) / 57.7 (94)	*χ*^*2*^ (1) = 0.15, *p =* 0.709
	DP	41.5 (170) / 38.7 (63)	*χ*^*2*^ (1) = 0.34, *p =* 0.558
	PA	16.8 (69) / 14.1 (23)	*χ*^*2*^ (1) = 0.61, *p =* 0.434
	ST	3.7 (15) / 2.5 [4]	*χ*^*2*^ (1) = 0.52, *p =* 0.472^**‡**^
Weekly therapy session (in the last 12 month), *M* (*SD*)		19.7 (8.4) / 17.7 (9.7)	*t* (264.43) = 2.33, *p* = 0.020*^§^

Note. CBT = cognitive behavioral therapy; DP = depth psychology, PA = psychoanalysis, ST = systemic therapeutic approach. *z* = standardized test statistic. ^**‡**^ Cell frequencies < 5, exact *p* value is calculated. ^**§**^ Levene-test with *p* = 0.005, adjusted test statistics is reported. *p < 0.05, **p < 0.01, ***p < 0.001

Table 4 shows responses of therapists with and without experience regarding their views on (a) consequences of climate change-related concerns for mental health, (b) how to address climate change-related concerns in therapy and (c) required resources for addressing climate change-related concerns in therapy. For the full range of responses on the original 4-point scale (from 1 = *I do not agree at all* to 4 = *I fully agree*) see Table B in Appendix B.

**Table 4:** Psychotherapists’ views on how to deal with climate change-related thoughts and feelings in therapy: comparison of psychotherapists with and without experience with respective patients

Items	Total % (n) (disagreement/ agreement)	With experience % (n) (disagreement/ agreement)	Without experience % (n) (disagreement/ agreement)	Group difference^‡^
**Views on consequences of climate change-related concerns for mental health**				
***In my opinion*** *…*				
…climate change-related thoughts and feelings have the potential to lead to serious **functional limitations in everyday life.**	27.5 (117) / 72.5 (308)	19.0 (51)/ 81.0 (217)	42.0 (66) / 58.0 (91)	*χ*^*2*^ (1) = 26.27, *p <* 0.001***
…climate change-related thoughts and feelings are **motivators to tackle climate change** and its consequences.	20.7 (88) / 79.3 (337)	15.3 (41) / 84.7 (227)	29.9 (47) / 70.1 (110)	*χ*^*2*^ (1) = 12.92, *p <* 0.001***
…climate change-related thoughts and feelings are **an expression of a zeitgeist.**	35.5 (150) / 64.5 (272)	41.4 (110) / 58.6 (156)	25.6 (40) / 74.4 (116)	*χ*^*2*^ (1) = 10.60, *p =* 0.002**
…climate change-related thoughts and feelings only become **relevant when they occur in the context of experienced traumatic events**, e.g., extreme weather events, natural disasters.	79.1 (334) / 20.9 (88)	87.6 (234) / 12.4 (33)	64.5 (100) / 35.5 (55)	*χ*^*2*^ (1) = 31.77, *p <* 0.001***
**Views on how to address climate change-related concerns in therapy**				
***In my opinion*** *…*				
…climate change related thoughts and feelings should be **taken up in a validating way** in therapy.	22.2 (93) / 77.8 (326)	18.2 (48) / 81.8 (216)	29.0 (45) / 71.0 (110)	*χ*^*2*^ (1) = 6.66, *p =* 0.030*
…climate change-related thoughts and feelings **can cause motivation and engagement that can be addressed in therapy** (e.g., self-efficacy, activity building, self-care).	15.2 (64) / 84.8 (358)	10.1 (27) / 89.9 (240)	23.9 (37) / 76.1 (118)	*χ*^*2*^ (1) = 14.43, *p <* 0.001***
…climate change-related thoughts and feelings can **lead to stress, resignation, or despair, which should be addressed therapeutically** (e.g., emotion regulation skills, relaxation, cognitive restructuring).	15.2 (64) / 84.8 (357)	12.0 (32) / 88.0 (234)	20.6 (32) / 79.4 (155)	*χ*^*2*^ (1) = 5.64, *p =* 0.036*
***I consider the topic important for my therapeutic work***, *…*				
…and I believe that **with the therapeutic skills I acquired, I am adequately prepared** to address patients’ climate change-related thoughts and feelings.	21.4 (89) / 78.6 (326)	20.8 (55) / 79.2 (210)	22.7 (34) / 77.3 (116)	*χ*^*2*^ (1) = 0.21, *p =* 0.648
…but I am **concerned about my own potential overload** from treating patients with climate change-related thoughts and feelings.	73.7 (306) / 26.3 (109)	69.1 (183) / 30.9 (82)	82.0 (123) / 18.0 (27)	*χ*^*2*^ (1) = 8.29, *p =* 0.016*
**Views on required resources for addressing climate change-related concerns in therapy**				
***I consider the topic important for my therapeutic work***, *…*				
…and **inform or educate myself** accordingly on how I can work therapeutically with patients expressing climate change-related thoughts and feelings.	46.3 (192) / 53.7 (223)	34.0 (90) / 66.0 (175)	68.0 (102) / 32.0 (48)	*χ*^*2*^ (1) = 44.64, *p <* 0.001***
…but **I don’t know where I can find information/further training** on how to deal with patients expressing climate change-related thoughts and feelings.	49.5 (205) / 50.5 (209)	46.4 (123) / 53.6 (142)	55.0 (82) / 45.0 (67)	*χ*^*2*^ (1) = 2.83, *p =* 0.092
I do **not consider the topic important** for psychotherapy and **I am not interested in further information** on how to work therapeutically with patients expressing climate change-related thoughts and feelings.	80.3 (335) / 19.7 (82)	89.5 (238) / 10.5 (28)	64.2 (97) / 35.8 (54)	*χ*^*2*^ (1) = 38.83, *p <* 0.001***

Note. Groups differ in experience with patients with climate change-related concerns. ^**‡**^*p-*values are corrected by Bonferroni-Holm correction [47]. *p < 0.05, **p < 0.01, ***p < 0.001

Therapists with experience approved significantly more often of all four statements regarding the potential consequences of climate change-related concerns to mental health. Around 80% (217/268) were convinced that climate change-related concerns can lead to serious functional impairment in everyday life. Whereby also more than half of the therapists without experience (58.0%, 91/157) agreed to this view. Therapists with experience (87.6%, 234/267) saw significantly more often relevant negative consequences to mental health, apart from traumatization e.g., due to extreme weather events. This opinion was also frequent in therapists without experience (64.5%, 100/155).

Regarding the views on how to address the topic in therapy, therapists with experience significantly more often agreed to the statement that climate change-related concerns should be taken up in a validating way (81.8%, 216/264). Additionally, therapists with experience are significantly more likely to approve of the statements that stress caused by climate change-related concerns (88.0%, 234/266) as well as motivation caused by climate change-related concerns (89.9%, 240/267) should be addressed in therapy. Both groups, therapists with (79.2%, 210/265) and without (77.3%, 116/150) experience reported having acquired adequate therapeutic skills to address climate change-related concerns in therapy and did not differ significantly in this regard. Around 30% (82/265) of therapists with experience stated concerns about their own potential overload from dealing with this topic in therapy. Also, nearly 20% (27/150) of therapists without experience stated the same concerns. There was a significant difference between the groups.

Regarding the views on required resources, therapists with experience significantly more often express an interest in educating and informing themselves. However, both groups of therapists reported difficulties in finding information or training on how to deal with patients expressing climate change-related concerns in therapy. Finally, therapists without experience significantly more frequently denied the importance of addressing the topic in therapy.

## Discussion

The current study investigated in a nationwide sample of psychotherapists in Germany whether they see patients with climate change-related concerns in their practice. We further examined characteristics of patients with such concerns and the therapists’ views on the topic of climate change in therapy. The results showed that the majority of therapists is already confronted with this topic in therapy. Although close to 80% of the therapists felt adequately prepared by applying their current therapeutic skills, half of them wished for more information and training on how to deal with such concerns in therapy.

The number of therapists (72%) indicating to see patients with climate change-related concerns in treatment found in this study is even higher than the number in the survey conducted with MHPs in the USA [24]. Hoppe, Prussia [24] reported that around 54% of MHPs stated to see clients with such concerns. However, since we exclusively sampled psychotherapists (opposed to the broader group of MHPs) who reported on their patients, it is likely that the patients discussed by the therapists in this study were heavily burdened. A review by Woodland, Ratwatte [9], comprising 31 studies, revealed an association between pre-existing mental health impacts and the exacerbation of mental health problems due to consequences of climate change (i.e., acute weather events). These findings may elucidate the higher proportion of therapists treating such patients, in contrast to the observations of Hoppe Hoppe, Prussia [24]. Furthermore, there is evidence suggestion an increased awareness of climate change in Germany in recent years [22] and compared to other nations [28]. The biennial Special Eurobarometer surveys on Climate Change conducted from 2009 to 2021 evaluated European perceptions of climate change, involving over 26,000 participants from the 27 EU member states and the UK. The results from 2019 indicated that more than eight out of ten respondents in Germany regarded climate change as a ‘very serious’ issue (81%), surpassing the EU average of 79% [28].

Further, 41% of the 410 therapists with experience stated that at least one patient with such concerns explicitly sought treatment because of these. This suggests that some patients seem to relate their functional impairment and distress to climate change and its consequences and seek treatment for this reason. This finding aligns with causal process diagram of Berry, Waite [4] to conceptualize relations between climate change and mental health. Climate change could influence mental health via various (in)direct paths. For example, the “loss of personal mental health resources” can be caused by an impaired capacity to cope with adversities and thus directly increase the risk of mental illness. Correspondingly, therapists in this study reported that their patients often experienced feelings of helplessness and frustration, which could be indicative of a reduced capacity to cope. In addition, climate change and its consequences could function as an additional stressor, increasing the mental health burden of patients. Together with other currently salient threats to the basis of existence, like the Ukraine war or sustained consequences of the Covid-19 pandemic [29] climate change-related concerns could add to pathology in a dose-effect manner.

The most frequently reported diagnoses of patients raising climate change-related concerns in this study were depression, adjustment disorder, and general anxiety disorder. According to available data of a Germany-wide research data platform (KODAP, short for the *coordination of data collection and evaluation at research and training outpatient clinics for psychotherapy*) containing the diagnoses of 4266 adult patients treated in 2016, the most frequent diagnoses defined as treatment causes were affective disorders (39.4%), of these 36% were depressive episode/disorder diagnoses. Anxiety disorders accounted for 14.2% of the index diagnoses, whereby generalized anxiety disorder took 14th place under the 50 most frequent diagnoses with 2.3%. Frequent index diagnoses were also adjustment disorder with 4.5% (6th most frequent given diagnosis) [30, 31]. Comparing the KODAP composition of diagnoses to our results implies that the three most frequent reported diagnoses are overrepresented in the present study. This could be related to differences in the settings. As KODAP provided data from patients in training outpatient clinics, we primarily surveyed psychotherapists about their patients in private practices. The current study cannot definitively determine whether these diagnoses occur more frequently in patients raising climate change-related concerns compared to other patients treated by the participants in their respective settings. Given the absence of comparative data, it is important to interpret these results with caution. Thus, it remains uncertain whether overrepresentation of these diagnoses in our study can be in any way linked to the presence of climate change-related concerns. Also, it needs to be stated that even strong emotional responses are part of an adequate reactions to the threat of climate change and can initiate an adaptive process [26, 32, 33]. However, climate change awareness could lead to extreme worrying, as several large-scale surveys have indicated [20–22]. The core symptom of generalized anxiety disorder is characterized by severe and persistent worrying [34] and thus concerns about consequences of climate change could act as such a core symptom. This is consistent with the results of the present study, as well as the survey of Hoppe, Prussia [24]. Both indicated that generalized anxiety disorder is frequently reported in relation to climate change mental health impacts. Yet, there is an ongoing debate about whether climate change-related symptoms are linked with established diagnoses (e.g., generalized anxiety disorder, as we considered above) or whether the broad range of climate change-related reactions indicates a need for an additional diagnostic category [4, 8, 32].

Furthermore, this study examined psychotherapists’ views on dealing with climate change-related concerns during treatment. Overall, both therapists with and without experience were convinced in the majority that climate change-related concerns have the potential to lead to serious functional impairment in patients and need to be taken up in a validating way in therapy. Therapists considered climate change-related concerns relevant for mental health, even when concerns were not related to traumatic experiences associated with climate change. This seems to indicate that psychotherapists have already consulted the currently existing literature on the acute and chronic effects of climate change consequences on mental health and is in line with our finding that more than the half of the participating psychotherapists undertake further information or training on these topics.

In addition, Budziszewska and Jonsson [23] conducted a qualitative study interviewing ten Swedish patients, who addressed climate change-related concerns within treatment. Results showed that an effective treatment (from the patients’ perspective) required psychotherapists’ knowledge about climate change and the competence to use this knowledge. This demands therapists to confront this topic themselves. In our study in more than 20% of the participating psychotherapists (more often with experience) worries occurred about a potential overload caused by treating patients with climate change-related concerns. This worry should be taken seriously in training and dissemination endeavors and indicates the need for self-care strategies and adequate supervision [8, 35, 36]. Nevertheless, almost 80% of respondents in this study felt well prepared to work with patients with climate change-related concerns using the therapeutic skills they had already acquired. The survey of Hoppe, Prussia [24] reported that less than a third of MHPs felt adequately prepared for this topic in treatment. As our study exclusively surveyed psychotherapists, our sample was likely to be trained more homogenously and specifically than the broad profession group (social workers, family and marriage counselors, psychologists) recruited by Hoppe, Prussia [24], which could explain the higher rate of preparedness found in our study. Nonetheless, our results are in line with findings of Gossmann, Rosner [37], who outlined in a German study surveying psychotherapists (*N* = 1358) about their work satisfaction, that psychotherapists in Germany felt efficacious, skillful, and able to deal with stressful situations in general.

There are several implications of the present findings for research and practice. Future studies are required to assess whether severe emotional reactions and high levels of functional impairment due to climate change-related concerns are related to specific established diagnoses or form climate change-specific pathologies. There is an increasing amount of literature seeking to comprehend psychological reactions to climate change consequences. More and more researchers aim to define and evaluate constructs and develop measures to get an exhaustive picture of climate change-related reactions and consequences for mental health [17, 19, 26, 38–41]. Moreover, our results indicate a lack of information about how to address climate change-related concerns in psychotherapy. There is already some guideline literature that covers tasks and challenges in the areas of research and practice and provides initial therapeutic considerations [8, 35, 36, 42, 43]. However, material for psychoeducation, guidelines, and components for treatment of climate change-related concerns as well as strategies for psychotherapists’ self-care could be improved by a continuous refinement of the conceptualization and knowledge of mental health impacts of climate change.

### Strengths and limitations

This study is - to the best of our knowledge - the first study assessing psychotherapists’ observations of patients raising climate change-related concerns and their views on dealing with this topic in therapy. A notable strength of this study lies in the composition of our sample of psychotherapists regarding the distribution of age, gender, practice setting and therapeutic approaches which was similar to the general population of psychotherapists practicing in Germany [44]. In the present study, approximately 60% of participating therapists fell within the age range of 36 to 61 years, with three-quarters being female. Official data for Germany indicated that 58% of employed psychotherapists fell within the age range of 35 to 60 years, with a female majority of 76.7% [44]. Furthermore, in our study, 79% of the psychotherapists practiced in private practice, while 11% were employed in hospital settings. Comparatively, official data reported that 71% worked in private practice and 15% were employed in hospitals [44]. Regarding therapeutic approaches, CBT was the most prevalent at 56.5%, followed by DP at 40.7%. PA at 16.3% and ST at 3.3%. Data from psychotherapists working with both adults and children/adolescents showed a similar distribution [45].”

However, there are several limitations. First, the presence of a self-selection bias cannot be discounted. It is plausible that therapists with a specific interest in climate change might have been more likely to participate in this study. Around 17% of respondents indicated involvement in advocacy groups, but the general level of involvement of German psychotherapists remains indeterminate due to a lack of comparative numbers. Secondly, the data on therapists’ diagnoses regarding climate change-related concerns are aggregated and retrospective estimates provided promptly during survey completion. The validity of these data needs to be treated with caution. Third, psychotherapists with the focus on adult patients were overrepresented in our study. This means that, this survey does not cover children and adolescents seeking treatment adequately. Further, it is important to note that the insights regarding patients’ and therapists’ awareness of climate change may not be generalized to other EU countries as Germany exhibits notably higher levels of climate change awareness compared to the EU average [46]. Final, the items were specifically developed for this study with no prior validation. Yet, the items were adapted from a large-scale survey with MHPs in the US [24] as we also aimed at descriptive results on the status quo on this topic in Europe.

## Conclusion

This study provides first findings on the presence of patients with climate change-related concerns in therapy in Europe. Psychotherapists generally considered the impact of climate change on their patients’ mental health to be significant to psychotherapeutic care. Further research is needed to explore the associations between these concerns and psychological symptoms as well as to develop effective interventions to address these concerns.

### Electronic supplementary material

Below is the link to the electronic supplementary material.

Supplementary Material 1: The complete survey is presented in Appendix A. The list of patients’ reaction is provided in Table A in appendix B and the full range responses of therapists’ views on the original 4-point scale (from 1 = *I do not agree at all* to 4 = *I fully agree*) is provided in Table B in Appendix B

## Data Availability

The data will be made available upon reasonable request from the authors.

## Abbreviations

CBT: Cognitive behavioral therapy
DP: Depth psychology
IPCC: Intergovernmental panel on climate change
KODAP: Coordination of data collection and evaluation at research and training outpatient clinics for psychotherapy
LPT: Licensed psychotherapist
MHP: Mental health professionals
PA: Psychoanalysis
PiT: Psychotherapist in training
PTSD: Posttraumatic stress disorder
ST: Systemic therapeutic approach
