# Correction: climate change-related concerns in psychotherapy: therapists’ experiences and views on addressing this topic in therapy

**DOI:** 10.1186/s40359-024-01783-w

**Published:** 2024-05-21

**Authors:** Katharina Trost, Verena Ertl, Julia König, Rita Rosner, Hannah Comtesse

**Affiliations:** Clinical and Biological Psychology, Catholic University Eichstaett-Ingolstadt, Eichstaett, Germany

BMC Psychology (2024) 12:192.

10.1186/s40359-024-01677-x.

Following publication of the original article, it came to the authors’ attention that that there was an error in the Data availability declaration of the article: the declaration said “No datasets were generated or analyzed during the current study”, whereas it should say “The data will be made available upon reasonable request from the corresponding author”. The declaration has been corrected in the published article.

