# Supplementary material for: Climate change-related concerns in psychotherapy: therapists’ experiences and views on addressing this topic in therapy
Source: BMC Psychol. 2024 Apr 8;12:192. doi: 10.1186/s40359-024-01677-x (PMC11003001; doi:10.1186/s40359-024-01677-x)
Supplement: Supplementary file 1 — Supplementary Material 1: The complete survey is presented in Appendix A. The list of patients’ reaction is provided in Table A in appendix B and the full range responses of therapists’ views on the original 4-point scale (from 1 = I do not agree at all to 4 = I fully agree) is provided in Table B in Appendix B [file 40359_2024_1677_MOESM1_ESM.docx]

**Appendix to**

***Climate change-related concerns in psychotherapy:***

***Therapists’ experiences and views on addressing this topic in therapy***

**Appendix A**

**Survey: Climate change-related thoughts and feelings in therapy**

**Please answer a few questions about yourself first:**

Gender: o m o f o d

Year of birth: ______(YYYY)

In which state do you currently reside? (List of federals states)

Your place of employment?

o Private practice

o Hospital

o Outpatient clinic

o Other: _______________

Since which year have you been working as a psychotherapist (including your time in training): _____(YYYY)

Indicate the number of therapy sessions you have carried out on average per week over the past 12 months:

Average number of sessions per week: _______

Please indicate, what applies to you:

You work as a (if applicable):

o Licensed Child and Adolescent Psychotherapist

o Licensed Psychotherapist for adults

o Licensed Psychotherapist for adults, with additional qualification for children and adolescents

o Physician psychotherapist

o Other

You are currently undergoing training as (if applicable):

o Psychotherapist for adults

o Psychotherapist for children and adolescents

o Psychotherapist for adults, additional qualification for children and adolescents

o Physician psychotherapist

o Other

Your therapeutic approach:

o Cognitive behavioral therapeutic approach

o Depth psychology

o Psychoanalysis

o Systemic therapeutic approach

o Other

Are you involved in climate change-related interest groups?

o no o yes

which one? _____

Do you act in an environmentally conscious manner in your daily life?

o In almost all areas of everyday life

o In many areas of everyday life

o In a few areas of everyday life

o In no area of everyday life

***Due to the increased events attributed to consequences of climate change (e.g., flood, drought periods), more and more people are becoming aware of the threat posed by climate change. Climate change-related thoughts and feelings, such as anger, fear, grief, and hopelessness, may arise in relation to perceived consequences and anticipated effects on both humans and nature.***

Have you already encountered patients in your work life who express climate change-related thoughts and feelings?

o yes o no

*If no:* I believe that in the future, I will see an increasing number of patients who express climate change-related thoughts and feelings in my psychotherapeutic work.

o yes o no

*If yes***: Questions for therapists with experience:**

***Please, now consider all the patients you have encountered in your therapeutic work over the past 12 months:***

What proportion of patients expressed climate change-related thoughts and feelings?

_____% percent in the last year. That's approximately ____ patients.

What proportion of these patients explicitly stated climate change-related thoughts and feelings as the reason for therapy?

0 ------------------------------------------------------------------------------------------------------- 100%

***Please now consider all the patients you have encountered throughout your entire therapeutic work to date:***

How many of the patients expressed climate change-related thoughts and feelings?

Please provide a number: ____ patients

***Please, now consider all these patients with climate change-related thoughts and feelings.***

Estimate the age of these patients (multiple answers possible):

o < 14 years (childhood)

o 14 to 18 years (youth)

o 19 to 24 years (young adulthood)

o 25 to 34 years

o 35 to 44 years (middle adulthood)

o 45 to 59 years old

o 60 to 70 years (old adulthood)

o > 70 years

Estimate the (targeted) educational qualification of these patients: (multiple answers possible)

o Basic school certificate

o Intermediate school certificate

o Higher education entrance qualification (A-Levels)

o University degree

Estimate the marital status that most of these patients had:

o majority of such patients is in a relationship

o majority of such patients is not in a relationship

o majority of such patients is too young for a relationship

Estimate whether these patients predominantly have children or not:

o majority of such patients have children

o majority of such patients do not have children

o majority of such patients are still a child themselves

Indicate the diagnosis/diagnoses you have assigned to these patients *(asked to sort by frequency)*

*To do this, use the numbers 1 to 5 according to the frequency of assignment: 1 corresponds to the most frequently assigned diagnosis, 2 to the second most frequently assigned diagnosis, etc. If you have given the same number of diagnoses to patients with climate change-related thoughts and feelings, you can use the same number several times. If you have not assigned one or more diagnoses, leave the fields blank.*

o Depression

o Adjustment Disorder

o Generalized anxiety disorder

o Panic disorder

o Somatoform disorders

o Posttraumatic stress disorder

o Agoraphobia

o Other: ___________

**The following lists climate change-related reactions that were common upon individuals expressing climate change-related emotional reactions.**

**Continue to consider all your patients with climate change-related thoughts and feelings. How were climate change-related thoughts and feelings expressed in therapy?**

Specify which cognitive styles you are aware of among your patients with climate change-related thoughts and feelings:

o Rumination:

if selected: What was the content? _______

o Catastrophic thoughts

if selected: What was the content? _______

o Other: _______

if selected: What was the content? _______

o Did not occur

Specify which emotions you are aware of among your patients with climate change-related thoughts and feelings: _____, _____,______,______,______

Specify whether or which physiological reactions occurred or were mentioned when it came to climate change-related thoughts and feelings:

o Racing heart

o Sweating

o Shortness of breath

o Sleep disorder

o Other: __________

o Did not occur

Specify whether or which behaviors occurred or were mentioned when it came to climate change-related thoughts and feelings:

o Crying

o Aggression or loud scolding

o Avoidance

o Other: __________

o Did not occur

**Questions for therapists with and without experience with patients expressing climate change-related concerns:**

**View on the importance of climate change-related thoughts and feelings in therapy**

***The following questions pertain to your view on the consequences of climate change-related thoughts and feelings on mental health, and how you think they should be dealt with in therapeutic practice. We are interested in your opinion, regardless of whether you have had concrete experiences with such patients.***

Please indicate the extent to which you agree with the following statement on the 4-point scale: *I do not agree at all– I rather disagree – I rather agree – I fully agree*

**In my opinion…**

…climate change-related thoughts and feelings have the potential to lead to serious functional limitations in everyday life.

…climate change-related thoughts and feelings are motivators to tackle climate change and its consequences.

…climate change-related thoughts and feelings are an expression of a zeitgeist.

…climate change-related thoughts and feelings only become relevant when they occur in the context of experienced traumatic events, e.g., extreme weather events, natural disasters.

…climate change related thoughts and feelings should be taken up in a validating way in therapy.

…climate change-related thoughts and feelings can cause motivation and engagement that can be addressed in therapy (e.g., self-efficacy, activity building, self-care).

…climate change-related thoughts and feelings can lead to stress, resignation, or despair, which should be addressed therapeutically (e.g., emotion regulation skills, relaxation, cognitive restructuring).

**I consider the topic important for my therapeutic work, …**

…and I believe that with the therapeutic skills I acquired, I am adequately prepared to address patients’ climate change-related thoughts and feelings.

…but I am concerned about my own potential overload from treating patients with climate change-related thoughts and feelings.

…and inform or educate myself accordingly on how I can work therapeutically with patients expressing climate change-related thoughts and feelings.

…but I don't know where I can find information/further training on how to deal with patients expressing climate change-related thoughts and feelings.

I do not consider the topic important for psychotherapy and I am not interested in further information on how to work therapeutically with patients expressing climate change-related thoughts and feelings.

**Appendix B**

**Table A**

Psychotherapist-reported cognitive, emotional, physiological and behavioral reactions of patients having expressed climate change-related concerns

| **Cognitive styles, % (*n*)** *n*^†^ = 316 (*multiple answers possible*) | |
| --- | --- |
| Rumination | 73.1 (231) |
| Catastrophic thoughts | 59.2 (187) |
| Other | 14.2 (45) |
| Did not occur | 5.4 (17) |
| **Feelings, % (*n*)** *n*^†^ = 219 (*multiple answers possible)* | |
| Anxiety | 88.6 (194) |
| Helplessness/ powerlessness | 60.7 (133) |
| Anger | 60.3 (132) |
| Grief/ sadness | 35.2 (77) |
| Frustration/ resignation | 16.0 (35) |
| Guilt/ shame | 8.7 (19) |
| Other | 7.8 (17) |
| **Physiological reactions, % (*n*)** *n*^†^ = 258 (*multiple answers possible*) | |
| Racing heart | 27.9 (72) |
| Sweating | 10.5 (27) |
| Shortness of breath | 11.2 (29) |
| Sleep disorder | 60.1 (155) |
| Other | 13.2 (34) |
| Did not occur | 31.8 (82) |
| **Behavioral reactions, % (*n*)** *n*^†^ = 262 (*multiple answers possible*) | |
| Crying | 34.0 (89) |
| Aggression or loud scolding | 44.7 (117) |
| Avoidance | 57.3 (150) |
| Other | 22.1 (58) |
| Did not occur | 16.4 (43) |

*Note*. *n***^†^ =** Number of all participants answering this item.

**Table B**

Views on how to deal with patients expressing climate change-related concerns in therapy listed by therapists’ experiences with such patients

|  | **Total** | | | | **Having experience** | | | | **Without experience** | | | |
| --- | --- | --- | --- | --- | --- | --- | --- | --- | --- | --- | --- | --- |
| **Items** | full refusal, % (*n*) | partially  refusal, % (*n*) | partially  agree-ment, % (*n*) | full agree-ment, % (*n*) | full refusal, % (*n*) | partially  refusal, % (*n*) | partially  agree-ment, % (*n*) | full agree-ment, % (*n*) | full refusal, % (*n*) | partially  refusal, % (*n*) | partially  agree-ment, % (*n*) | full agree-ment, % (*n*) |
| **Views on consequences of climate change-related concerns for mental health** | | | | | | | | | | | | |
| **In my opinion …** | | | | | | | | | | | | |
| …climate change-related thoughts and feelings have the potential to lead to serious **functional limitations in everyday life.**  (*n_total_* = 425, *n_wE_* = 268) | 9.9 (42) | 17.6 (74) | 45.6 (194) | 26.8 (114) | 6.0 (16) | 13.1 (35) | 51.9 (139) | 29.1 (78) | 16.6 (26) | 25.5 (40) | 35.0 (55) | 22.9 (36) |
| …climate change-related thoughts and feelings are **motivators to tackle climate change** and its consequences.  (*n_total_* = 425, *n_wE_* = 268) | 7.1 (30) | 13.6 (58) | 57.4 (244) | 21.9 (93) | 2.2 (6) | 13.1 (35) | 64.2 (172) | 20.5 (55) | 15.3 (24) | 14.6 (23) | 45.9 (72) | 24.2 (38) |
| …climate change-related thoughts and feelings are **an expression of a zeitgeist.**  (*n_total_* = 422, *n_wE_* = 266) | 16.4 (69) | 19.2 (81) | 43.6 (184) | 20.9 (88) | 17.3 (46) | 24.1 (64) | 42.1 (112) | 15.5 (44) | 14.7 (23) | 10.9 (17) | 46.2 (72) | 28.2 (44) |
| …climate change-related thoughts and feelings only become **relevant when they occur in the context of experienced traumatic events**, e.g., extreme weather events, natural disasters.  (*n_total_* = 422, *n_wE_* = 267) | 59.0 (249) | 20.1 (85) | 17.3 (73) | 3.6 (15) | 68.9 (184) | 18.7 (50) | 11.2 (30) | 1.1 (3) | 41.9 (65) | 22.6 (35) | 27.7 (43) | 7.4 (12) |
| **Views on how to address climate change-related concerns in therapy** | | | | | | | | | | | | |
| **In my opinion…** | | | | | | | | | | | | |
| …climate change related thoughts and feelings should be **taken up in a validating way** in therapy.  (*n_total_* = 419, *n_wE_* = 264) | 9.5 (40) | 12.6 (53) | 35.3 (148) | 42.5 (178) | 6.1 (16) | 12.1 (32) | 39.0 (103) | 42.8 (113) | 15.5 (24) | 13.5 (21) | 29.0 (45) | 41.9 (65) |
| …climate change-related thoughts and feelings **can cause motivation and engagement that can be addressed in therapy** (e.g., self-efficacy, activity building, self-care).  (*n_total_* = 422, *n_wE_* = 267) | 5.2 (22) | 10.0 (42) | 43.6 (184) | 41.2 (174) | 2.2 (6) | 7.9 (21) | 45.3 (121) | 44.6 (119) | 10.3 (16) | 13.5 (21) | 40.6 (63) | 35.5 (55) |
| …climate change-related thoughts and feelings can **lead to stress, resignation, or despair, which should be addressed therapeutically** (e.g., emotion regulation skills, relaxation, cognitive restructuring). (*n_total_* = 421, *n_wE_* = 266) | 5.7 (24) | 9.5 (40) | 41.1 (173) | 43.7 (184) | 3.4 (9) | 8.6 (23) | 44.4 (118) | 43.6 (116) | 9.7 (15) | 11.0 (17) | 35.5 (55) | 43.9 (68) |
| **I consider the topic important for my therapeutic work, …** | | | | | | | | | | | | |
| …and I believe that **with the therapeutic skills I acquired, I am adequately prepared** to address patients’ climate change-related thoughts and feelings.  (*n_total_* = 415, *n_wE_* = 265) | 5.5 (23) | 15.9 (66) | 48.9 (203) | 29.6 (123) | 4.9 (13) | 15.8 (42) | 50.6 (134) | 28.7 (76) | 6.7 (10) | 16.0 (24) | 46.0 (69) | 31.3 (47) |
| …but I am **concerned about my own potential overload** from treating patients with climate change-related thoughts and feelings. (*n_total_* = 415, *n_wE_* = 265) | 51.3 (213) | 22.4 (93) | 23.1 (96) | 3.1 (13) | 44.5 (118) | 24.5 (65) | 28.3 (75) | 2.6 (7) | 63.3 (95) | 18.7 (28) | 14.0 (21) | 4.0 (6) |
| **Views on required resources for addressing climate change-related concerns in therapy** | | | | | | | | | | | | |
| **I consider the topic important for my therapeutic work, …** | | | | | | | | | | | | |
| …and **inform or educate myself** accordingly on how I can work therapeutically with patients expressing climate change-related thoughts and feelings.  (*n_total_* = 415, *n_wE_* = 265) | 21.9 (91) | 24.3 (101) | 38.8 (161) | 14.9 (62) | 10.6 (28) | 23.4 (62) | 45.7 (121) | 20.4 (54) | 42.0 (63) | 26.0 (39) | 26.7 (40) | 5.3 (8) |
| …but **I don't know where I can find information/further training** on how to deal with patients expressing climate change-related thoughts and feelings. (*n_total_* = 414, *n_wE_* = 265) | 27.5 (114) | 22.0 (91) | 31.9 (132) | 18.6 (77) | 24.5 (65) | 21.9 (58) | 35.5 (94) | 18.1 (48) | 32.9 (49) | 22.1 (33) | 25.5 (38) | 19.5 (29) |
| I do **not consider the topic important** for psychotherapy and **I am not interested in further information** on how to work therapeutically with patients expressing climate change-related thoughts and feelings.  (*n_total_* = 417, *n_wE_* = 266) | 66.4 (277) | 13.9 (58) | 11.5 (48) | 8.2 (34) | 77.1 (205) | 12.4 (33) | 9.0 (24) | 1.5 (4) | 47.7 (72) | 16.6 (25) | 15.9 (24) | 19.9 (30) |

*Note.* *n_total_* = number of answering participants in total, *n_wE_* = number of answering participants with experience.
